# Artificial intelligence of stenosis on coronary CTA: real world comparison with quantitative coronary angiography

**DOI:** 10.1093/ehjimp/qyaf152

**Published:** 2025-11-29

**Authors:** Sharjeel Hassan, Christina Mansour, Megan Pelter, Livia De Sousa Domingues Da Silva, Eric Hu, Chih-Wei Chang, Alexander R Van Rosendael, Melody Hermel, Elizabeth Epstein, Samantha Bagsic, Sanjeev Bhavnani, Austin Robinson, Shawn Newlander, Jorge Gonzalez, Andrew Chiou, Keshav Nayak, George Wesbey

**Affiliations:** Department of Medicine, Scripps Mercy Hospital, San Diego, CA, USA; Department of Medicine, Scripps Mercy Hospital, San Diego, CA, USA; Department of Cardiology, Scripps Clinic, La Jolla, CA, USA; Department of Cardiology, Scripps Clinic, La Jolla, CA, USA; Department of Cardiology, Scripps Clinic, La Jolla, CA, USA; Department of Cardiology, Scripps Clinic, La Jolla, CA, USA; Department of Cardiology, Scripps Clinic, La Jolla, CA, USA; Department of Cardiology, Scripps Clinic, La Jolla, CA, USA; Department of Cardiology, Scripps Clinic, La Jolla, CA, USA; Department of Cardiology, Scripps Clinic, La Jolla, CA, USA; Department of Cardiology, Scripps Clinic, La Jolla, CA, USA; Department of Cardiology, Scripps Clinic, La Jolla, CA, USA; Department of Cardiology, Scripps Clinic, La Jolla, CA, USA; Department of Cardiology, Scripps Clinic, La Jolla, CA, USA; Department of Cardiology, Scripps Clinic, La Jolla, CA, USA; Department of Medicine, Scripps Mercy Hospital, San Diego, CA, USA; Department of Cardiology, Scripps Clinic, La Jolla, CA, USA; Department of Cardiology, Scripps Clinic, La Jolla, CA, USA

## Introduction

Recent advances in artificial intelligence (AI)-driven quantitative coronary computed tomography angiography (AI-QCT) have shown promise in the non-invasive assessment of coronary artery disease (CAD). Prior trials, including the CREDENCE substudy by Griffin *et al*. demonstrated that AI-based coronary CTA can closely approximate results from blinded core-lab quantitative coronary angiography (QCA) for vessels >2.0 mm.^1^ However, there remains a critical need to validate these findings in real-world clinical settings, particularly with variable image quality and patient heterogeneity. This study aimed to evaluate the diagnostic accuracy of AI-QCT against invasive QCA using real-world, single-center data. Our analysis incorporated all assessable vessels ≥1.5 mm in QCA diameter and focused on per-territory agreement to reflect practical applicability.

## Methods

We conducted a retrospective analysis of 88 consecutive stable, adult patients between 1 June 2016 through 21 March 2018 who underwent clinically indicated 256-slice volumetric single-heart beat coronary CTA, followed within 90 days by invasive coronary angiography (ICA). 70 patients with non-revascularized native coronary vessels were included. 18 patients with prior percutaneous coronary intervention (PCI) with stent placement or coronary artery bypass grafting (CABG) were excluded. There were no exclusions for image quality for either CCTA or ICA. Age, sex, BMI, and Agatston coronary calcium score were collected. CCTA scans from the site-selected best phase of the cardiac cycle were analyzed using CLEERLY, an FDA-approved AI-based software platform capable of automated quantitative plaque and stenosis assessment. All coronary territories were included in the analysis, and all evaluable coronary vessels ≥1.5 mm in diameter were assessed. A total of 14 out of 998 coronary segments (1.4%) were excluded due to non-diagnostic or poor image quality. QCA from the invasive catheterization was performed by a senior interventional cardiologist blinded to both CCTA images and reports and ICA reports, with diameter and percent stenosis measurements obtained using commercial FDA-approved QCA software. The primary outcomes were detection of ≥50% and ≥70% stenosis, assessed both at the per-territory and per-patient levels. Per-territory and per-patient diagnostic performance metrics, including sensitivity, specificity, positive predictive value (PPV), negative predictive value (NPV), accuracy, and area under the curve (AUC), were calculated for each threshold.

## Results

The final analysis included 207 coronary territories from 70 patients. Mean age was 64.2 ± 10.7 years, with 32.9% female, average BMI 28.4 ± 6.1 kg/m², and 25.7% obese. Mean Agatston calcium score was 639.1 ± 773.9. For detection of ≥50% stenosis per territory, AI-QCT demonstrated a sensitivity of 0.71, specificity of 0.75, PPV of 0.53, NPV of 0.87, overall accuracy of 0.74, and an AUC of 0.83. For ≥70% stenosis per territory, performance improved, with sensitivity of 0.78, specificity of 0.91, PPV of 0.51, NPV of 0.97, accuracy of 0.89, and an AUC of 0.89. On a per-patient basis for detecting ≥50% stenosis, AI-QCT showed a sensitivity of 0.86, specificity of 0.43, PPV of 0.69, NPV of 0.67, accuracy of 0.69, and AUC of 0.77. At the ≥70% threshold, sensitivity was 0.86, specificity 0.79, PPV 0.66, NPV 0.93, accuracy 0.81, and AUC 0.91.

**Figure 1:**
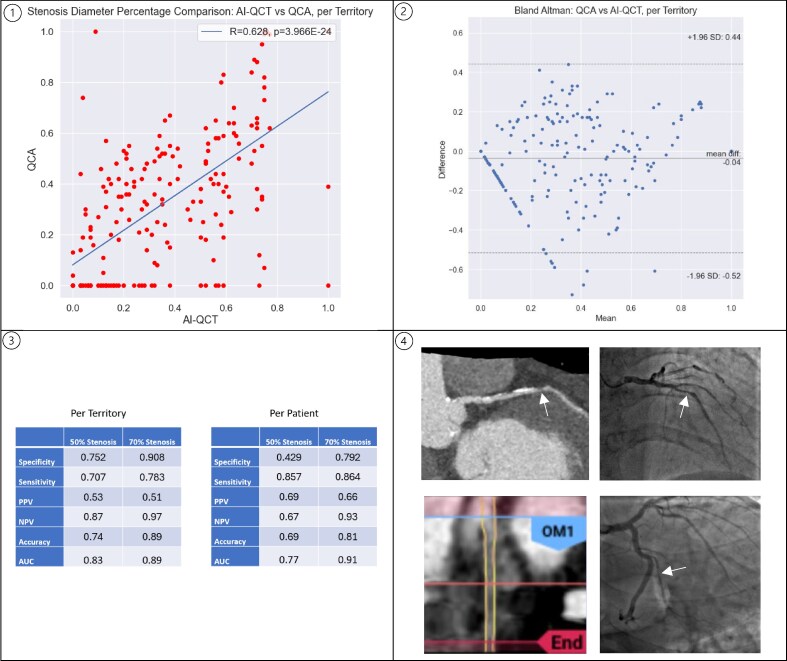
Representative visual summary of diagnostic performance and validation metrics for AI-QCT vs. QCA. (1) Scatter plot showing correlation between AI-QCT and invasive QCA for percent diameter stenosis measurements on a per-territory basis. (2) Bland-Altman plot assessing agreement between AI-QCT and QCA per-territory stenosis measurements. (3) Tables showing diagnostic classification performance of AI-QCT for detecting ≥50% and ≥70% stenosis, stratified by both per-territory and per-patient analysis. (4) Representative FN cases on AI-QCT. Top row: LAD non-calcified lesion not detected by AI-QCT. Onsite-generated curved reformat (left) and ICA with 1.92 mm proximal lumen diameter and 70% stenosis by QCA (right), with white arrow indicating stenosis. FFR was 0.77, stent was placed. Bottom row: Proximal OM1 lesion not detected on AI-QCT straightened lumen view (left) and ICA view with white arrow highlighting the 74% stenosis by QCA with proximal lumen diameter 1.82 diameter (right). No stent placed.

Error analysis revealed that false positives occurred in 10 patients for 70% stenosis. Conversely, false negatives were noted in 3 territories in 3 patients for 70% or more stenosis, all in vessels < 2 mm, all with non-calcified plaques (*Figure 1*). To assess agreement between modalities, scatter and Bland-Altman plots were generated for per-territory stenosis measurements (*Figure 1*).

## Conclusion

In this single-center, real-world validation study, AI-QCT demonstrated strong diagnostic performance for identifying coronary artery stenosis, particularly at the ≥70% threshold. Its high NPV and favorable AUC support its potential role as a non-invasive gatekeeper to ICA. We selected both ≥50% and ≥70% thresholds to reflect common clinical decision points, ≥50% as a marker of potentially flow-limiting disease in research and guideline definitions, and ≥70% as a threshold more consistently associated with ischemia and used to guide revascularization decisions. This dual-threshold approach also allowed us to assess performance across the spectrum of clinically relevant stenosis severity.

However, the PPV was only moderate, most notably for the ≥50% threshold (per-territory PPV 0.53), underscoring the need for careful clinical interpretation to avoid overestimating stenosis severity and triggering unnecessary invasive procedures. This limitation was less pronounced at the ≥70% threshold, where specificity and AUC improved substantially, but clinicians should remain mindful of false positives, especially in small-caliber vessels and heavily calcified segments.

Compared with the CREDENCE substudy by Griffin *et al*. our study included coronary segments down to 1.5 mm in diameter (vs. ≥2 mm), a range that approaches the spatial resolution limits of conventional CT scanners.^2^ This inherent technical constraint may have contributed to reduced accuracy in smaller vessels and helps explain some of the observed discrepancies in per-territory performance, including false negatives with underestimation of stenosis severity.^2^

As a retrospective analysis, our findings are subject to inherent limitations including potential selection bias and reporting bias. While we attempted to mitigate these through inclusion of consecutive cases and blinding of the invasive interpretation, residual confounding cannot be excluded.

We did not perform a direct comparison between AI-QCT stenosis or AI-QCT ischemia and invasive physiology assessments (i.e. adenosine-FFR), as only a small portion of patients underwent physiologic testing, limiting statistical power. Of the 10 false-positive segments at the ≥70% stenosis threshold, 5 had invasive pressure measurements performed at ICA, and 4 demonstrated ischemia in the corresponding segments. We have previously reported the accuracy of AI-QCT ischemia relative to adenosine FFR in a different real-world cohort.^3^

Given the high mean Agatston score (639 ± 774) in our cohort, calcification burden likely contributed to false positives by overestimating luminal stenosis. Although subgroup analysis by calcium score was not performed due to sample size constraints, this limitation should be considered when interpreting AI-QCT in heavily calcified populations.

Despite these challenges, AI-QCT maintained high diagnostic utility across thresholds and analysis levels, reinforcing its value in real-world settings. These findings underscore the importance of external validation in diverse patient populations, where broader inclusion criteria and variable image quality more accurately reflect clinical practice, and support the integration of AI-QCT into CAD evaluation workflows to improve triage and reduce unnecessary invasive testing.

## Lead author biography


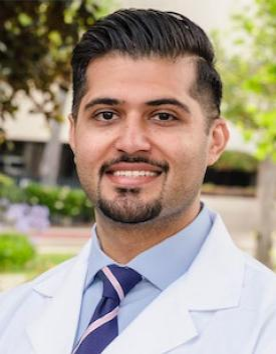


Dr Sharjeel Hassan is the Chief Internal Medicine Resident at Scripps Mercy Hospital in San Diego, CA, USA. He earned his Doctor of Osteopathic Medicine degree from the Burrell College of Osteopathic Medicine in New Mexico. He completed his internal medicine residency at Scripps Mercy, where he now serves as Chief Resident. His academic interests include non-invasive cardiovascular imaging, lipidology, and personalized approaches to cardiovascular risk assessment. He plans to pursue a career in cardiology with a focus on imaging and prevention.

## Data Availability

Data will be available to share with interested investigators through reasonable request with the study PIs, following appropriate data sharing agreements consistent with institutional policies and complying with IRB standards.
